# Prevalence and diversity of H9N2 avian influenza in chickens of Northern Vietnam, 2014

**DOI:** 10.1016/j.meegid.2016.06.038

**Published:** 2016-10

**Authors:** Duong Mai Thuy, Thomas P. Peacock, Vu Thi Ngoc Bich, Thomas Fabrizio, Dang Nguyen Hoang, Nguyen Dang Tho, Nguyen Thi Diep, Minh Nguyen, Le Nguyen Minh Hoa, Hau Thi Thu Trang, Marc Choisy, Ken Inui, Scott Newman, Nguyen vu Trung, Rogier van Doorn, Thanh Long To, Munir Iqbal, Juliet E. Bryant

**Affiliations:** a National Center for Veterinary Diagnostics, Department of Animal Health, Hanoi, Vietnam; b Avian Viral Diseases programme, The Pirbright Institute, Woking, UK; c St Mary's Campus, Imperial College London, London, UK; d Oxford University Clinical Research Unit and Wellcome Trust Major Overseas Programme, Hanoi, Vietnam; e St Jude's Center for Excellence in Influenza Research and Surveillance, Memphis, TN, USA; f Division of Epidemiology, Department of Animal Health, Hanoi, Vietnam; g MIVEGEC (UM1-UM2-CNRS 5290-IRD 224), Centre de Recherche IRD, Montpellier, France; h Food and Agriculture Organization of the United Nations, Hanoi, Vietnam; i National Hospital Tropical Diseases, Hanoi, Vietnam; j Center for Tropical Medicine, Nuffield Department of Clinical Medicine, University of Oxford, Oxford, UK

**Keywords:** LPAI, low pathogenicity avian influenza, HPAI, highly pathogenic avian influenza, FAO, Food and Agriculture Organization of the United Nations, LBM, live bird market, Avian influenza, Vietnam, H9N2, Poultry, Chicken, H5, Zoonotic

## Abstract

Despite their classification as low pathogenicity avian influenza viruses (LPAIV), A/H9N2 viruses cause significant losses in poultry in many countries throughout Asia, the Middle East and North Africa. To date, poultry surveillance in Vietnam has focused on detection of influenza H5 viruses, and there is limited understanding of influenza H9 epidemiology and transmission dynamics. We determined prevalence and diversity of influenza A viruses in chickens from live bird markets (LBM) of 7 northern Vietnamese provinces, using pooled oropharyngeal swabs collected from October to December 2014. Screening by real time RT-PCR revealed 1207/4900 (24.6%) of pooled swabs to be influenza A virus positive; overall prevalence estimates after accounting for pooling (5 swabs/pools) were 5.8% (CI 5.4–6.0). Subtyping was performed on 468 pooled swabs with M gene Ct < 26. No influenza H7 was detected; 422 (90.1%) were H9 positive; and 22 (4.7%) were H5 positive. There was no evidence was of interaction between H9 and H5 virus detection rates. We sequenced 17 whole genomes of A/H9N2, 2 of A/H5N6, and 11 partial genomes. All H9N2 viruses had internal genes that clustered with genotype 57 and were closely related to Chinese human isolates of A/H7N9 and A/H10N8. Using a nucleotide divergence cutoff of 98%, we identified 9 distinct H9 genotypes. Phylogenetic analysis suggested multiple introductions of H9 viruses to northern Vietnam rather than in-situ transmission. Further investigations of H9 prevalence and diversity in other regions of Vietnam are warranted to assess H9 endemicity elsewhere in the country.

## Highlights

• We report detection of highly pathogenic avian influenza (HPAI) from *healthy* chickens in Live Bird Markets of Vietnam. Because all breeds of domestic chickens are extremely susceptible to HPAI, we speculate that HPAI detections from market chickens may reflect infections that occur after arrival in the market. Alternatively, shedding of HPAI from healthy birds may reflect vaccine-induced protective immunity that mitigates disease but does not block viral infection.
• As many as 49% of all pooled surveillance swabs were positive for influenza A virus, corresponding to an overall Influenza A prevalence of 5.45% (95% Confidence Interval 5.4-6.0%).
• Low pathogenicity avian influenza (LPAI) H9N2 accounted for the vast majority of all influenza A detections in market chickens sampled from 9 northern provinces.
• To date there is no evidence to suggest an interaction effect between circulation of H5 and H9 viruses; however sampling strategies that involve pooling of surveillance swabs from multiple birds greatly complicates the assessment of co-infection rates or evaluation of epidemiological associations.

## 1 Introduction

Vietnam has a well-developed system for the active surveillance of highly pathogenic avian influenza (HPAI) strains within live bird markets (LBMs). From 2010 to 2015, surveillance focused primarily on monitoring genetic diversity and ongoing evolution of HPAI H5 subtype viruses within duck populations (Nguyen et al., 2014). Starting in 2013, additional surveillance activity was initiated in provinces along the Chinese border to promote early detection of H7N9 viruses, and was focused largely on collections from chickens and environmental samples. To date, this second surveillance activity has screened > 33,480 pooled chicken oropharyngeal swabs and environmental samples, however no A/H7N9 viruses have been identified in Vietnam (FAO, 2014).

Low pathogenicity avian influenza (LPAI) H9N2 is the most prevalent influenza subtype circulating endemically in chickens worldwide. H9N2 viruses are endemic in poultry across Asia, the Middle East and Northern Africa (Fusaro et al., 2011), and despite their status as LPAI, poultry outbreaks of H9N2 are associated with significant economic losses, largely due to reduced egg production, reduced feed conversion efficiencies, and highly lethal bacterial or viral co-infections (Li et al., 2005, Parvin et al., 2014). Epidemiological evidence indicates that the chicken adapted H9N2 genotype 57 (G57), also known as genotype S, contains an internal gene cassette (polymerase genes, nucleoprotein, matrix and non-structural genes) that confers increased host range and zoonotic transmission potential to non-H9N2 influenza strains, with recent examples including the zoonotic H7N9 and H10N8 outbreaks in China (H. Chen et al., 2014, Gu et al., 2014, Pu et al., 2014). Additionally H9N2 viruses pose a zoonotic risk in their own right, having caused sporadic human infections in China, Hong Kong, Bangladesh and Egypt (ICDDRB, 2011, Butt et al., 2005, Huang et al., 2015a, Huang et al., 2015b, WHO, 2015a, WHO, 2015b). It is suggested that the prevalence and variation of H9N2 influenza virus in farmed poultry could provide an important early warning of the emergence of novel reassortants with pandemic potential (Liu et al., 2014a, Liu et al., 2014b). Despite increasingly widespread use of H9 vaccines in neighboring China (Pu et al., 2014, Wei et al., 2016; P. Zhang et al., 2008), the poultry sector in Vietnam has not yet adopted any vaccination against H9N2 viruses, largely because poultry production remains dominated by smallholder farms, with little vertical integration and relatively low investment in veterinary vaccines. The only licensed avian influenza vaccines for poultry in Vietnam are inactivated virus vaccines against the H5 subtype (Nguyen et al., 2014).

Research and surveillance activities in Vietnam from 2003 to 2013 previously identified a number of LPAI subtypes circulating in duck populations (Hotta et al., 2012, Jadhao et al., 2009, Kim et al., 2013; D. C. Nguyen et al., 2005, Nomura et al., 2012; Masatoshi Okamatsu et al., 2013, Takakuwa et al., 2013), including all 3 major antigenically divergent lineages of LPAI H9N2 (i.e. the Chinese BJ94/Y280-like lineage, the Middle Eastern G1-like lineage and the predominantly Korean Y439-like lineage) (Jadhao et al., 2009, Kim et al., 2013, Nomura et al., 2012). In contrast, LPAI transmission within chickens has not been well studied. Here we determine the prevalence and diversity of LPAI in chickens from northern Vietnam, and evaluate the feasibility of direct sequencing of AIV from pooled surveillance swabs.

## 2 Methods

### 2.1 Sample collections, molecular screening, and virus isolations

Samples used in this study were collected at 10 time points from October to December 2014, representing one discrete period of surveillance activity in the FAO-supported programme for early detection of H7N9 viruses. This surveillance activity focused on sampling LBM in northern provinces believed to play a key role in cross-border poultry trade with China. The sampling frame was based on a stratified sampling design, and entailed collections in 7 northern provinces, with a primary focus on market trade routes along the Vietnam/China border. Five district markets were sampled at weekly intervals for approximately 10 weeks between October 30 and December 31 of 2014. At each sampling timepoint, oropharyngeal (OP) swabs of 35 chickens were sampled individually from 7 different vendors per market, and aggregated into pools (5 swabs per pool). The pooled OP swabs were stored in 2 mL viral transport medium (PBS pH 7.2 supplemented with antibiotics, gentamicin, and buffering agents), transported by cold chain to the National Center for Veterinary Diagnostics (NCVD) and stored at -80 °C prior to further processing. RNA of pooled swab samples were extracted using a taco^TM^ Nucleic Acid Automatic Extraction System (GeneReach Biotechnology Corp., Taiwan). Detection of influenza A and subtype-specific monoplex real time RTPCR were performed using standardized protocols (Supplementary T2). Allantoic fluid from inoculated embryonated chicken eggs were clarified by centrifugation for 10 min at 4 °C, and presence of virus was determined by hemagglutination assay as previously described (WHO, 2011).

### 2.2 Next generation sequencing (NGS)

We used NGS protocols for influenza sequencing adopted from St Jude's Center for Excellence in influenza Research & Surveillance (SJCEIRS). Briefly, fresh nucleic acid extracts were prepared and samples were pre-amplified to enrich for influenza genomes using MBTUni12/13 primers as previously described (Zaraket et al., 2015, Zhou et al., 2009). DNA libraries were prepared using Nextera XT DNA Library Prep Kits (Illumina) with 96 dual-index barcodes according to manufacturer's instructions. Pooled libraries were sequenced on a MiSeq Illumina, using the v2 reagents and 300 cycles. Sequence reads were filtered for quality scores, and the high quality reads aligned to a custom reference library (kindly provided by SJCEIRS) and by *de novo* assembly. Genomic sequences and variant frequencies were generated in CLC genomics workbench v. 8.5.1 (http://www.clcbio.com/).

### 2.3 Phylogenetic and genotypic analysis

Data used for phylogenetic analyses comprised the novel whole genome sequences generated by this study, as well as additional publicly available sequences of closely related viruses identified by Blastn analysis of NCBI and GISAID databases. Full datasets for each gene segment were aligned using the MUSCLE algorithm in Mega software version 6.06 (www.megasoftware.net). Alignments were trimmed to yield the maximal sequence coverage per segment as follows: 260-2289 for PB2; 78-2242 for PB1; 232-1981 for PA; 160-1599 for HA; 49-1507 for NP; 24-1386 for NA; 26-996 for MP; and 56-859 for NS. Phylogenetic trees were created using the neighbor-joining algorithm and the Tajima-Nei model, with 1000 bootstrap replications. Bootstrap values of < 70 were excluded from trees. Genotyping was performed using 2% nucleotide difference cutoff to indicate genetically divergent segments.

### 2.4 Statistical analysis

Prevalence of influenza type A, H5 and H9 virus infection was estimated from the pooled samples at the market and province levels, using a maximum likelihood modeling approach described in Supplementary Appendix A. We used an influenza A Matrix gene Ct threshold of 35 as the standard cut-off for positivity. For subtype specific prevalence of H5 and H9, the threshold for positivity was set at Ct ≤ 38. Evidence for interaction between H9 and H5 from pooled samples was tested by a likelihood ratio test as described in the Supplementary Appendix B.

### 2.5 Nucleotide accession numbers

The complete and partial genome sequences of the viruses sequenced in this study were submitted to the GenBank database (Table S2).

## 3 Results

### 3.1 Prevalence of influenza A in market chickens

**Fig. 1:**
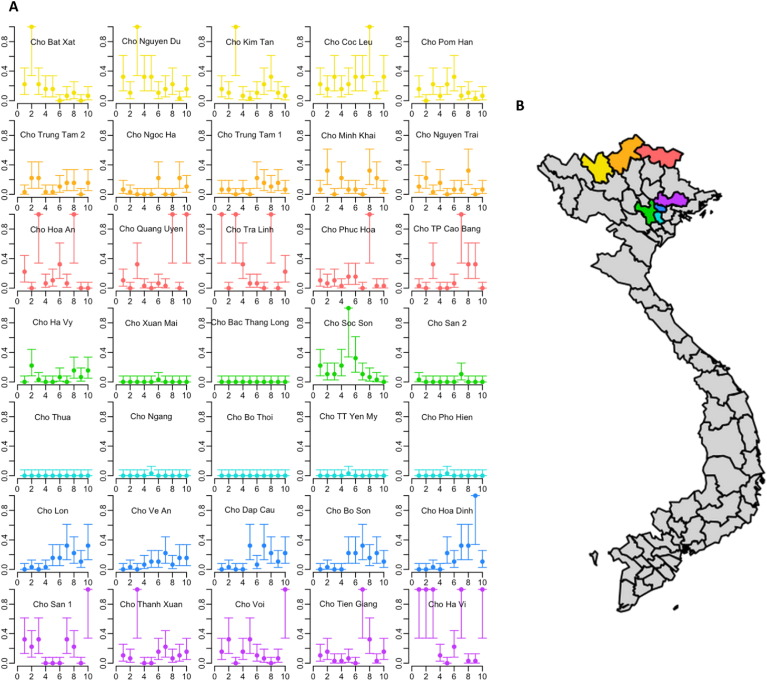
(a) Influenza A prevalence estimates in 5 district markets each for 7 northern provinces from Oct.–Dec. 2014; (b) map showing provinces surveyed by the H7F programme. Yellow = Lao Cai province; orange = Ha Giang province; red = Cao Bang province; green = Ha Noi province; purple = Bac Giang province.

A total of 1207 of 2450 pooled swabs (49%) screened positive for influenza A by matrix gene real time RT-PCR. Using a maximum likelihood modeling approach that accounts for pooling (Suppl Appendix A), this detection level corresponded to an overall influenza A prevalence of 5.45% (95% Confidence Interval [CI] 5.4–6.0%). Fig. 1(a) presents estimated prevalence of influenza A per weekly sampling round (x-axis), depicted by province (rows) and by market (individual graphs). None of the 1207 influenza positive pools were found positive for H7 subtype. Further molecular subtyping to detect H5 and H9 viruses was conducted on all pooled swabs with an influenza A matrix gene Ct < 26 (n = 468) (Table 1). Detection rates for H9 and H5 subtype varied dramatically between provinces and individual markets, with H9 accounting for 64–100% of influenza A positive pools; H5 was detected in 4.7% (22/468) of positive pools; 3.8% (18/468) pools screened positive for both H5 and H9 subtypes; and 8.9% (42/468) were classified as ‘subtype unknown’. The maximum likelihood prevalence (accounting for pooling) for H9 and H5 was 3.7% (95%CI 3.3–4.1%) and 1.09% (95%CI 0.7–1.6%), respectively. We found no evidence of significant interaction between H5 and H9 when using a maximum likelihood modeling approach (Suppl Appendix B). Virus isolation in embryonated eggs was attempted on selected representative H9 positive pools with Ct < 24 (n = 25), and all H5 positive pools and pools the were ‘unknown subtype’, regardless of Ct value (n = 22 and 42, respectively). We isolated 25 H9 viruses, however none of the H5 positive pools or the pools of ‘unknown subtype’ yielded a virus isolate.

### 3.2 Phylogenetic analysis and genotyping

**Fig. 2:**
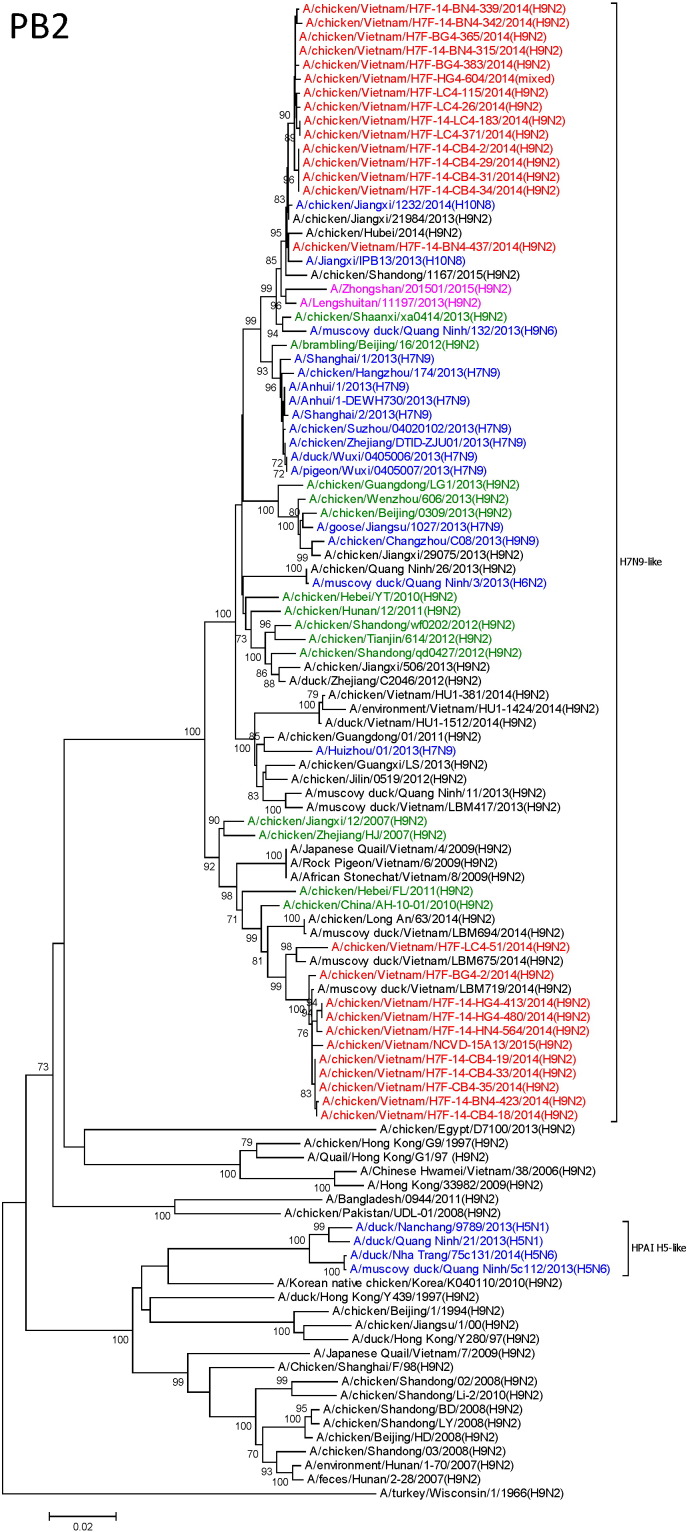

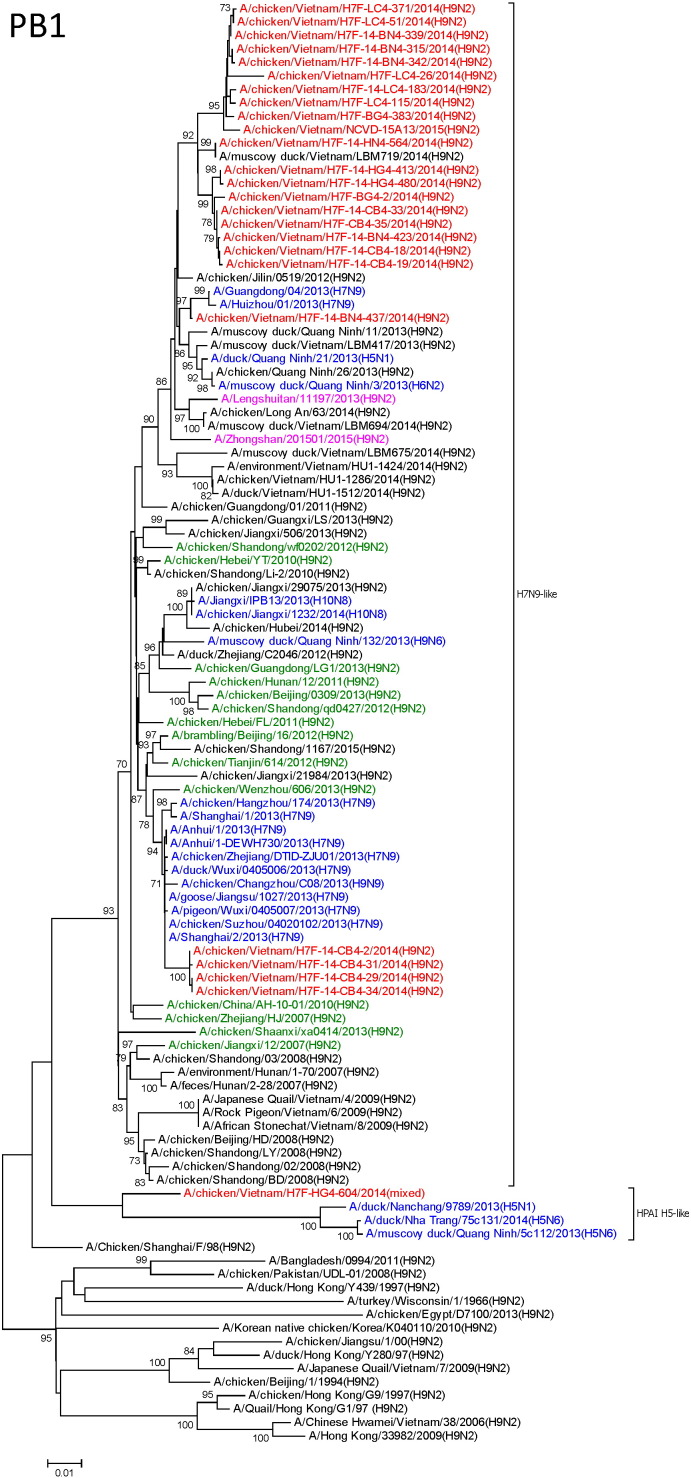

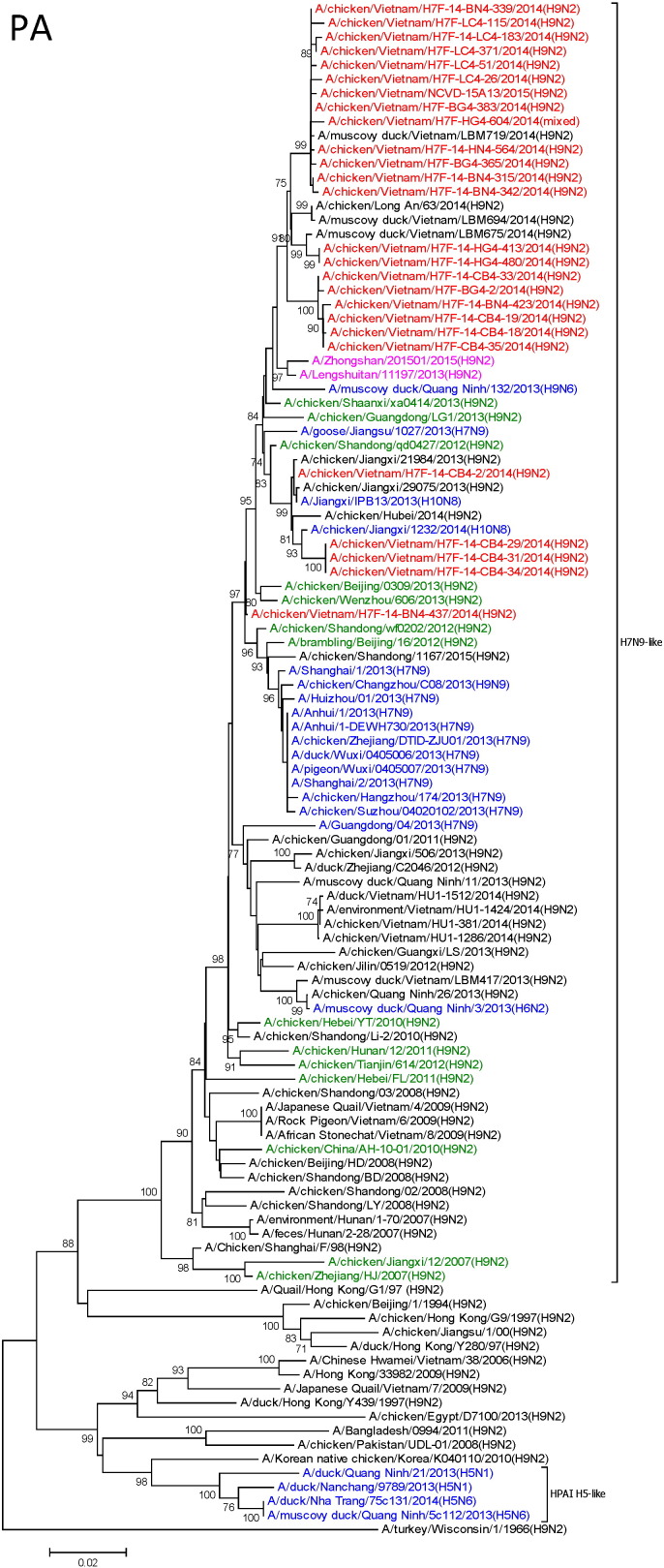

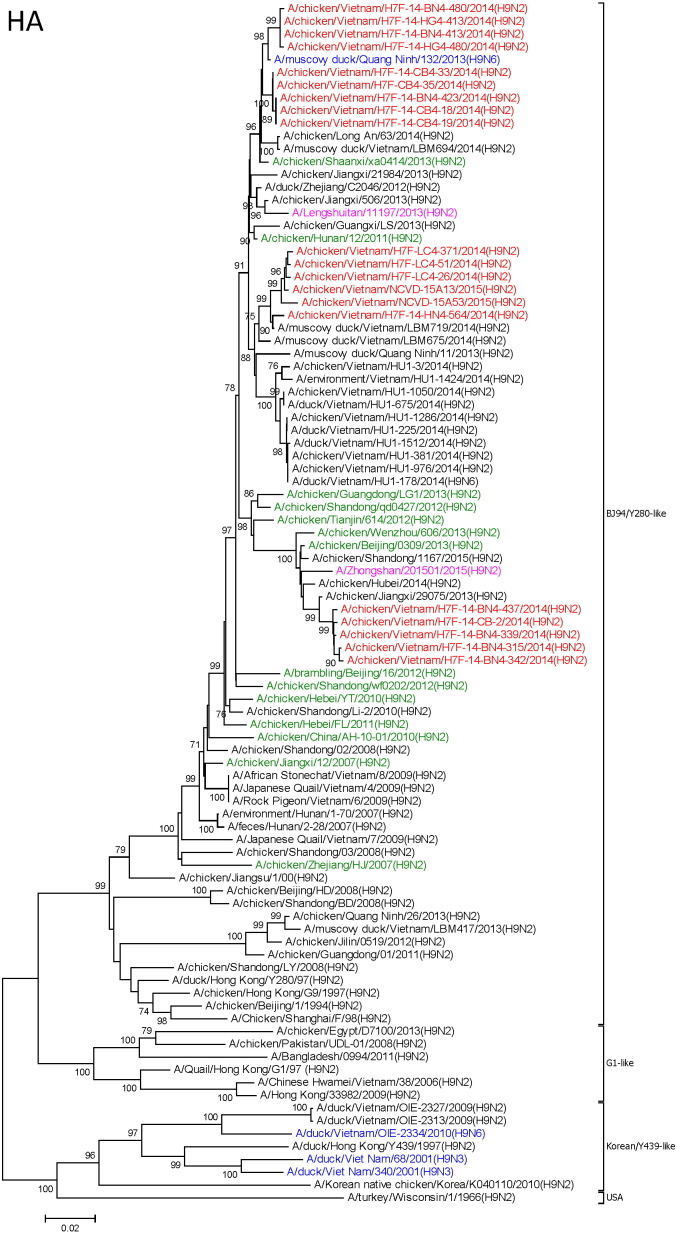

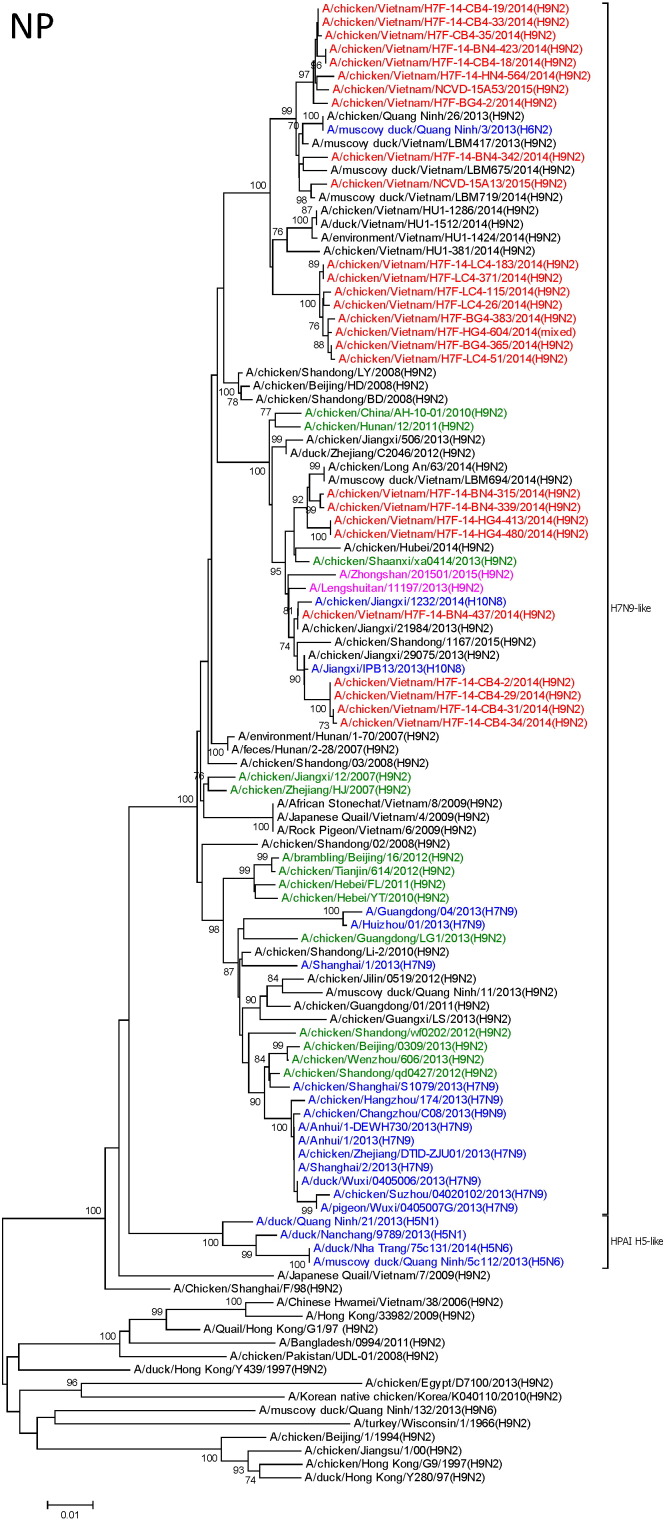

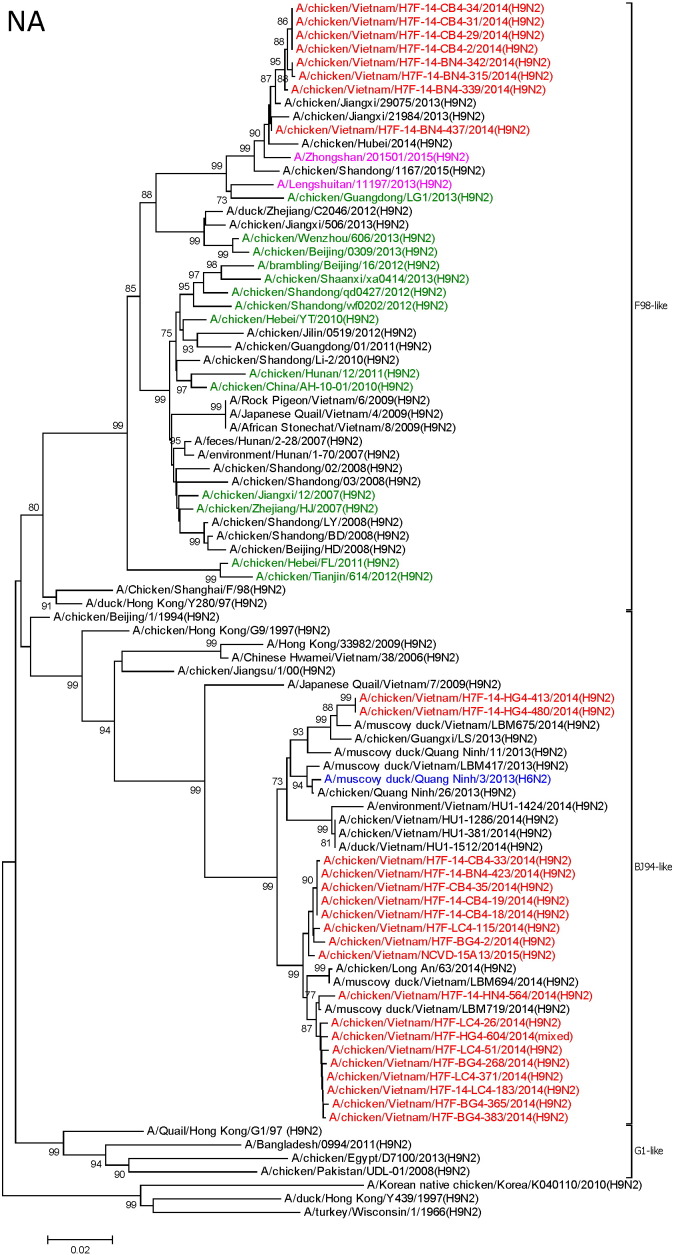

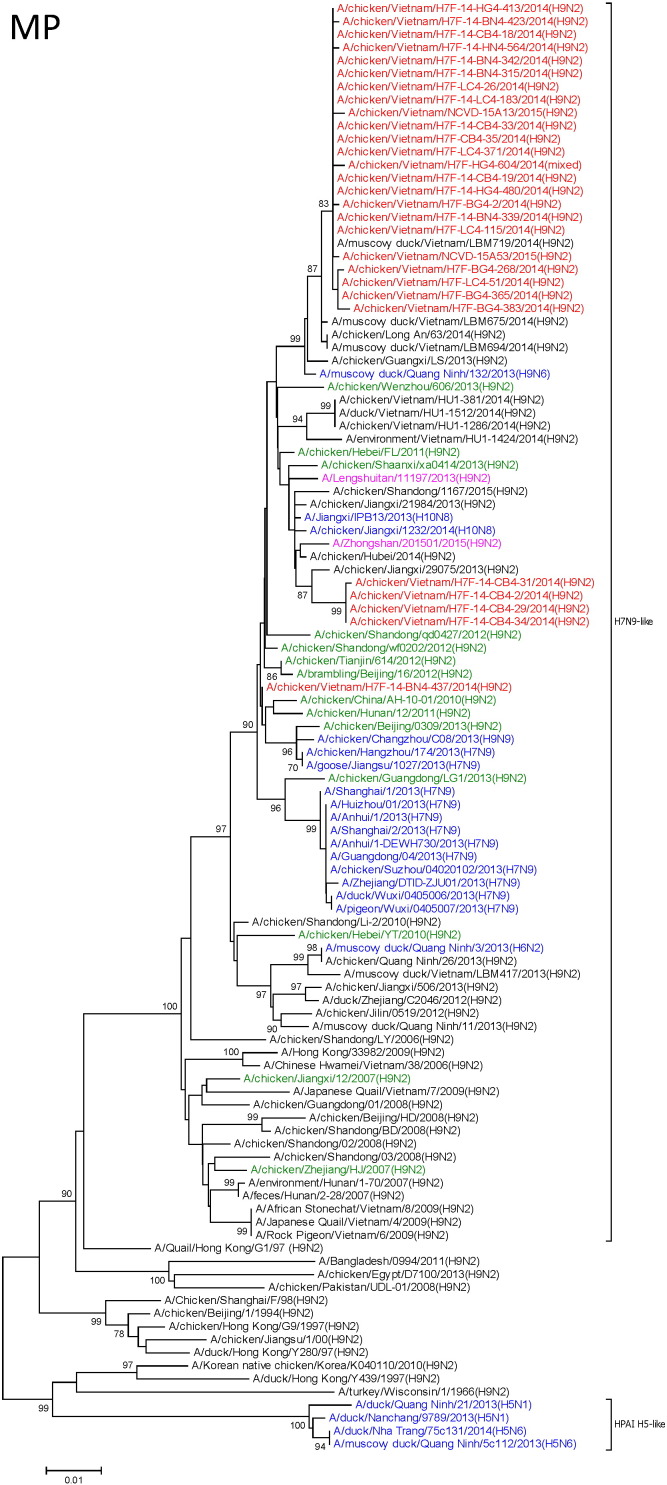

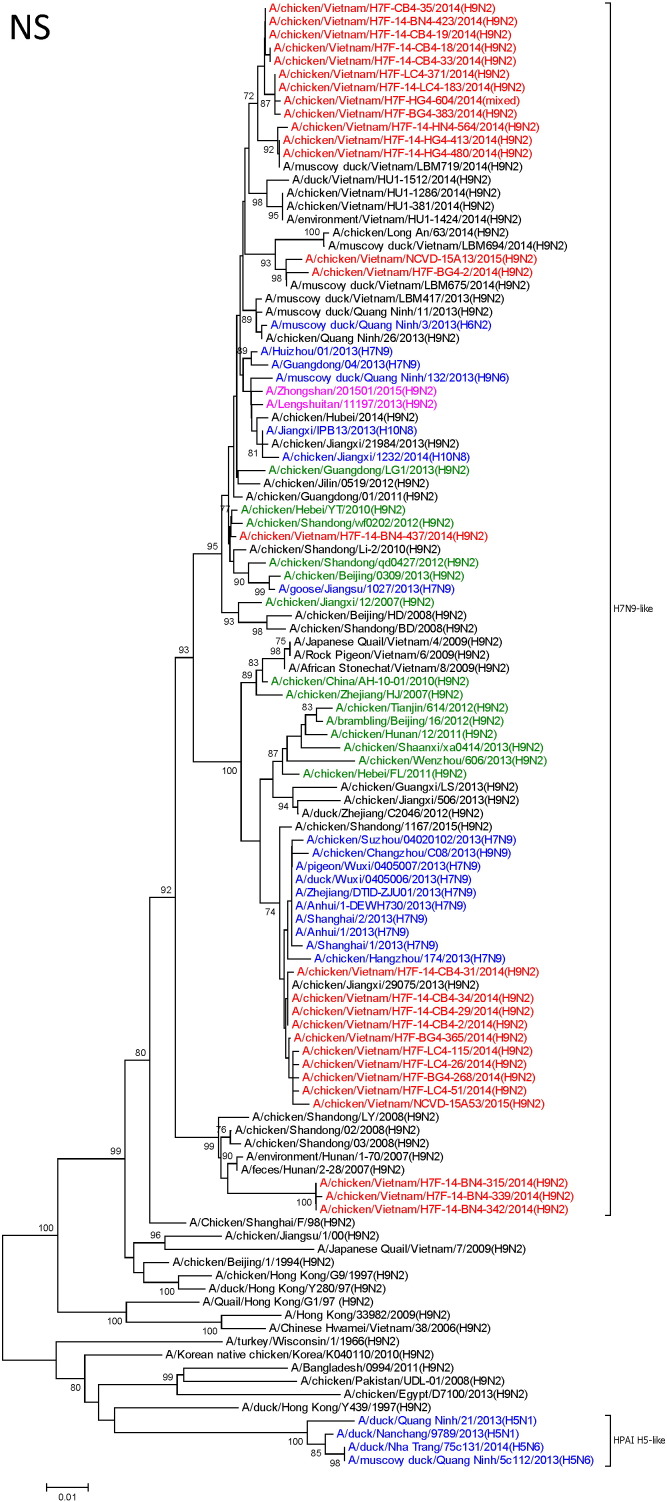
Neighbor joining phylogenetic trees of Vietnamese H9N2 viruses (n = 17) and reference strains (n = 83 strains). Red = virus sequences generated in this study; Green = genotype 57 viruses as defined by Pu et al., PNAS 2015; Blue = H9 sequences from non-H9N2 subtype viruses; Pink = recent genotype 57 human H9N2 isolates from China; Black = additional representative H9N2 viruses available from Genbank. Bootstrap values below 70% excluded.

We utilized next generation sequencing using the Illumina Nextera XT Sample Preparation Kit and the MiSeq platform as previously described (Zaraket et al., 2015) using both direct sequencing of pooled surveillance swabs, as well as egg-grown virus isolates. Amplification enrichment for influenza genomes was performed on a total of 96 samples (19 egg isolates, 77 pooled surveillance swabs), from which 55 were selected for Nextera XT library preparations, and 50 were included in the run. Next generation sequencing identified 17 H9N2 complete genomes, 2 H5N6 complete genomes, 11 partial genomes. The HPAI H5 genomes detected from LBM will be presented within a separate publication and are only briefly discussed here. To determine relationships among the novel A/H9N2 sequences, we performed Blastn on each gene segment to identify closely related viruses within public datasets, and included a number of additional references. All H9 HA genes fell into the BJ94/Y280-like lineage, the predominant H9 lineage found in China. Segment 6 (NA) exhibited the most diversity with 15.7% nucleotide differences between strains, and fell into 2 distinct clusters (Fig. 2). In contrast, the other segments showed reduced diversity, with maximum pairwise nucleotide differences of 6.0%, 4.0%, 2.9%, 5.0%, 4.8%, 1.6% and 6.0% for segments 1–5, 7 and 8 respectively.

**Fig. 3:**
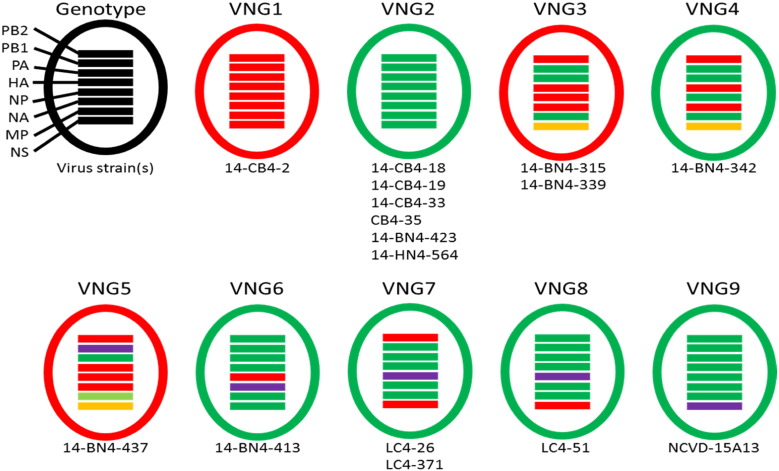
Genotype constellations for H9N2. Segments of different colours represent > 2% nucleotide difference. Different genotypes were assigned an arbitrary ‘Vietnam genotype #’ (VNG#).

Each of the 17 whole genomes of H9N2 possessed the G57-like internal gene cassette, similar to zoonotic H7N9 and H10N8 viruses in mainland China. For example, the genes most closely related to the PB2 and PB1 segments of H7F-14-BN4-437 belonged to the human H10N8 and H7N9 isolates, A/Jiangxi/IPB13/2013(H10N8) and A/Guangdong/04/2013(H7N9) with percentage nucleotide homologies of 99.6% and 99.5% respectively. Additionally H7F-14-CB4-2, H7F-14-CB4-29, H7F-14-CB4-31 and H7F-14-CB4-34 possessed PB1 and NS genes closely related to human and avian H7N9 isolates from 2013 with homologies of over 99.9%, and their PA, NP and MP genes were related to H10N8 viruses with homologies > 99.3%. Using an > 98% nucleotide difference cutoff for each segment, we determined patterns of clustering at the sub-genotype level, and identified 9 distinct sub-genotypes, all of which appeared to be reassortants between closely related H9N2 viruses (Fig. 3). (For reference, the comparable divergence level used for clade designations in H5 viruses is 1.5%) (WHO/OIE/FAO, 2012). Previous investigations of nucleotide substitution rates of H9N2 viruses have suggested that 2% divergence signifies approximately 4.5 to 9 years of evolution (Fusaro et al., 2011).

Of the 18 surveillance swabs initially identified by RT-PCR as containing both H5 and H9, only 8 were subjected to MiSeq (the other 10 samples were excluded during quality control steps), and 1 yielded sequence results indicating presence of both subtypes (H7F-CB4-35). Additionally, there was one sample that tested positive for H5 but not H9 on initial RTPCR screening (H7F-HG4-604), however it yielded H9HA, H5HA, H9-like PB2, PA, NP, NA, MP and NS genes, and an H5-like PB1. The status of these samples as intersubtypic reassortants cannot be established as they represented pooled swabs from multiple birds. Of the samples designated as ‘influenza positive, unknown subtype’ by RT-PCR, 3 of 9 samples sequenced by MiSeq yielded partial sequence data of H9N2, and the remaining did not yield sequences of sufficient coverage for analysis.

### 3.3 Molecular characteristics of the novel H9N2 genomes

#### 3.3.1 Hemagglutinin (HA)

**Fig. 4:**
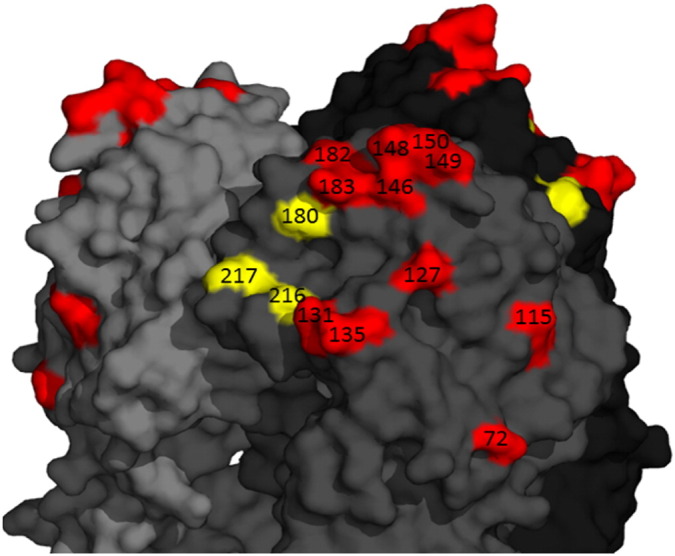
Structural diagram showing antigenically important amino acid substitutions observed in H9HA. Made using pymol, amino acids mapped onto the HA structure of A/Swine/Hong Kong/9/98 (H9N2) PDBID:1JSD.

The 19 H9 sequences generated retained the classical BJ94/Y280-like ‘low-pathogenic’ dibasic RSSR cleavage motif that has previously shown to allow cleavage by epithelial proteases such as matriptase and is thought to facilitate systemic infections in poultry (Baron et al., 2013). The receptor binding site of all sequences had leucine at position 216 (226 in H3 numbering), a residue associated with increased binding to human-like α2,6 linked sialic acids in multiple subtypes including H9N2, and consequently associated with mammalian adaptation and transmission (Sorrell et al., 2009). This is a change from previously detected Vietnamese H9N2 viruses which tended to have a glutamine at this position which preferentially binds to the ‘avian’ α2.3 linked sialic acid (Jadhao et al., 2009). Position 217 (227, H3 numbering) has been implicated in both receptor binding and mammalian adaptation in a number of subtypes, however the significance of methionine at this position (as observed in the H9 Vietnamese sequences here) remains unknown (Sang et al., 2015, Stevens et al., 2006). Additionally position 180 (190 H3 numbering) is implicated in receptor binding specificity in the closely related H1 hemagglutinins (Matrosovich et al., 2001, Matrosovich et al., 2000) the Vietnamese viruses have either alanine or valine, which are very common in recent Chinese H9s, or threonine, which is more rarely found, at this position. The HA sequences also contained a number of previously described markers with the potential to confer mammalian transmission potential, as well as the chicken airborne transmission marker K363 (Rudneva et al., 2005; Kaverin et al., 2005; Sun et al., 2013, Zhong et al., 2014a, Zhong et al., 2014b) (Table S1). All viruses contained conserved N-linked glycosylation sequence (N-X-S/T, where X =/=P) at positions 11–13, 123–125, 280–282, 287–289 and 295–297, and more than half of the sequences had an additional glycosylation sequence at position 200–202 (Table S1). Finally, by analyzing known H9N2 antigenic residues from the literature (Kaverin et al., 2004, Okamatsu et al., 2008, Peacock et al., 2016, Ping et al., 2008, Wan et al., 2014, Zhu et al., 2015), we predict there are at least two antigenically variant sub-groups circulating in northern Vietnam: those antigenically similar to H7F-14-CB4-2 and those similar to HF7-LC4-26 which vary from each other at 11 antigenically important residues (Fig. 4).

#### 3.3.2 Neuraminidase (NA)

The N2 genes of the Vietnamese viruses fell into two distinct clades (Fig. 2) within the G57 lineage, clade 0 and 1 (Pu et al., 2014). These distinct clades vary in regards to a 3 amino acid deletion in the NA stalk (clade 1 NAs have the deletion, clade 0 do not). This deletion, although minor compared to the 20 + amino acid deletions found recently in the N1 of HPAI H5N1 viruses, has been shown experimentally to increase the pathogenicity of H9N2 viruses in chickens and mice, and has become predominant in Chinese H9N2 circulation (94.8% of viruses since 2013) (Sun et al., 2013). Although the Vietnamese NA genes had a number of additional molecular markers of virulence and airborne transmission in mammals and poultry (Lv et al., 2015; Z. Zhang et al., 2011) (Table S1), they did not have any known molecular markers of oseltamivir resistance, and thus probably remain susceptible to the drug (Gubareva, 2004).

#### 3.3.3 NS1 and NEP

Similar to divergence of NA, segment 8 also segregated phylogenetically into two groups within the G57-like lineage, with a major group represented by H7F-14-CB4-2 and a minor group represented by H7F-14-BN4-315 (Fig. 2). Interestingly, the minor group contained a NS1 C-terminal elongation from 217 to 237 amino acids. This elongation has been shown to influence inflammation in poultry and mammalian experimental models, and to increase transmissibility among poultry (Kong et al., 2015). Similar to the Chinese human H9N2 isolates, the NS1 proteins of these virus contain a large number of mammalian markers (Dankar et al., 2011, Ping et al., 2011; Z. Zhang et al., 2011) (Table S1).

#### 3.3.4 Accessory proteins (PB1-F2, PA-X)

In addition to the 10 well characterized and almost universally expressed core proteins of influenza, some virus strains also code for accessory proteins such as PB1-F2 and PA-X, which are thought to be important in modulating virulence (Chen et al., 2001, Gao, 2015, Jagger et al., 2012). All sequenced PA segments coded for a complete PA-X protein, shown to increase mammalian replication and pathogenicity in H9N2 viruses (Gao, 2015). Length of the PB1-F2 protein, however, varied between different isolates. Of the H9N2-like PB1s, 18 isolates contained a full length PB1-F2 (90aa), 5 had a minor truncation (76aa), and a single isolate had a major truncation (27aa). 76aa long PB1-F2s from H9N2 viruses has previously been implied to increase the virulence of H7N9 viruses in mice but are thought not to interfere with function (Gibbs et al., 2003, Su et al., 2015). PB1-F2 functional knockouts, similar to the 27aa variant seen, have also been shown to increase virulence of avian influenza viruses (Gibbs et al., 2003). The PB1-F2 of the mixed isolate H7F-HG4–604 was H5-like and had a length of 57 amino acids, which like the 27aa variants is often considered a functional knockout (Gibbs et al., 2003). All PB1-F2s of over 76aa also had several molecular markers associated with mammalian virulence and predisposition to secondary bacterial pneumonia (Alymova et al., 2014) (Table S1).

#### 3.3.5 Polymerase subunits and nucleoprotein

The polymerase subunits of each of the H9N2 isolates, PB2, PB1, PA, and NP, displayed multiple mammalian adaptation markers (Chen et al., 2006, Mei et al., 2016, Park et al., 2015, Sun et al., 2013, Xiao et al., 2016, Yao et al., 2013) (Table S1). In all sequenced isolates the PA subunit had a marker of airborne transmission in chickens, L672 (Zhong et al., 2014a, Zhong et al., 2014b). However, none of the viruses had any of the polymerase markers most well associated with mammalian adaptation of avian influenza viruses, i.e. PB2 E627K or D701N (Herfst et al., 2012, Imai et al., 2012, Mehle and Doudna, 2009, Subbarao et al., 1993).

#### 3.3.6 Matrix proteins

The M1 and M2 proteins, encoded as alternative splice products of segment 7 of the influenza genome, similarly to the other segments, contained a number of known markers of mammalian adaptation (Song et al., 2009, Yao et al., 2013, Zhang et al., 2011) (Table S1). All viruses had an asparagine (N) at amino acid position 31 of M2, a mutation associated with resistance to antivirals amantadine and rimantadine (Belshe et al., 1988).

## 4 Discussion

Our results confirm the hyperendemicity of avian influenza viruses in chickens sampled in LBM of northern Vietnam, where influenza H9N2 viruses are by far the dominant subtype in circulation. Our detection rates are comparable to those reported from China, where overall prevalence of AIV in chickens has ranged from 21 to 36% in Hubei, 34–46% in Zhejiang province (Chen et al., 2016), and 15.7% in Hunan (Huang et al., 2015a), and where H9N2 is the dominant AIV subtype detected throughout all provinces (Shi et al., 2014). Although a plethora of AIV subtypes are known to circulate in wild and domestic Vietnamese ducks, including H3, H4, H5, H6, H7 and H10 viruses (Hotta et al., 2012, Jadhao et al., 2009; D. C. Nguyen et al., 2005, Nomura et al., 2012, Okamatsu et al., 2013, Takakuwa et al., 2013), our deep sequencing data confirm that the only detectable AIV subtypes circulating endemically within chickens are H5 and H9. Additionally, although no Chinese-like H7N9 viruses have presently been found in Vietnam, the H9N2 viruses sequenced here shared the H7N9-like G57/genotype S internal gene cassette (Gu et al., 2014, Pu et al., 2014). This internal gene cassette is thought to contribute to the pathogenicity of H7N9 viruses in mammals, to confer zoonotic and poultry enzootic potential to other subtypes (e.g. H10N8), and is at least partly responsible for the extremely high transmissibility of H9N2 viruses in chickens (Pu et al., 2014, Zhang et al., 2014). The H9N2 viruses circulating in northern Vietnam also closely resembled recent H9N2 strains isolated from human clinical cases in China (Huang et al., 2015b). Like its more temperate neighbor, China, Vietnam has seasonal fluctuations in AIV prevalence, with peak incidence in HPAI the winter months of November to February (Durand et al., 2015, Nguyen et al., 2014), and a similar pattern for LPAI (Pu et al., 2014).

Although the current surveillance system provides an excellent opportunity to monitor diversity of co-circulating HPAI H5 and LPAI H9N2, the process of combining swabs from multiple birds (i.e. pooling) complicates the inference of co-infection rates (and definitely decrease its power), and complicates analysis of intersubtypic reassortment. Among the deep sequencing data generated directly from surveillance swabs, although we could not assess *inter*subtypic reassortment between H5 and H9N2 viruses, the genomic analysis suggested high levels of *intra*subtypic reassortment between different subclades of H9N2 (Liu et al., 2014a, Liu et al., 2014b, Sun et al., 2010).

The genomic data demonstrated that most gene segments were closely related to Chinese viruses, which likely reflects proximity of our sampling sites to the Chinese-Vietnam border. Importation of live chickens from China has been illegal since May 2013; hence, these findings suggest that despite regulations to curtail illegal traffic in live poultry, there remains potential for cross-border spread. The pattern of phylogenetic clustering of the H9N2 sequenced here revealed two distinct clusters for each gene segment (or sometime 3 clusters, as in the case of the HA phylogeny), suggesting there may have been separate introduction events. However, H9N2 viruses are known to be highly transmissible between chickens and wild terrestrial birds (e.g. sparrows) (Iqbal et al., 2013), and thus cross-border wild bird migration may also have played a role in geographical spread of H9 viruses. Interestingly, the N2 sequences generated here were predominantly from the BJ94-like lineage, and, unlike all other gene segments, were most similar to Vietnamese N2s that have been circulating in Vietnam for at least a decade suggesting the possibility of underlying continual, *in situ* evolution of H9N2 viruses within Vietnam.

Given the evidence that prevalence and diversity of AIV in neighboring China continues to increase at alarming rates (Chen, 2016; Huang 2015), and given that Vietnam ranks as one of the countries with highest clade diversity for HPAI H5 (Shi et al., 2014), the public health concerns regarding potential adaptation of AIV to human transmission remain as pressing today as ever. Yet the numbers of recorded human cases of HPAI H5 in Vietnam are extremely low (total of 127 cases since 2003, and no cases recorded for 2015) (WHO, 2015b). High levels of ‘silent’ circulation of HPAI H5 in ducks have been well documented for many years (Nguyen et al., 2014), and are known to play a key role in endemic transmission ecology. In contrast, however, ‘silent’ shedding of HPAI H5 from apparently healthy chickens may be a relatively recent phenomenon. Asymptomatic shedding of HPAI H5 from chickens could be explained by multiple factors, with at least four possible scenarios: 1) the lack of symptoms may indicate subtle shifts in virulence associated with reassortant genomes (i.e. emergence of attenuated phenotypes); 2) the lack of symptoms could be attributed to use of poultry H5 vaccines that successfully mitigate disease symptoms of H5 infections, but fail to completely block virus replication and shedding; 3) the lack of symptoms may be related to prior heterologous immunity to H9N2, which has been shown to reduce HPAI disease severity but does not impact transmission (Khalenkov et al., 2009, Seo and Webster, 2001); or 4) the chickens that are detected as H5 positive may have become infected after arrival in the market, in which case the lack of symptoms (and the relatively low viral loads) could reflect the stage of infection (early phase incubation). Further examination of these questions would be facilitated by more extensive and systematic testing for both H5 and H9 within the surveillance system, and improved data collection for epidemiological risk factors (i.e. trace-back data on vaccination status, time to sale in market), and modeling approaches to assess overall transmission dynamics of H5 and H9 within the local context of Vietnam.

Regarding the human health implications of LPAI H9N2 circulation, previous studies of human seroprevalence conducted in northern Vietnam in 2001 documented HI titers to H9 and presence of H9 neutralizing antibodies in 3–4% of people tested, but with no significant differences between the general population and those with occupational exposures to poultry (Uyeki et al., 2012). We speculate that overall H9 prevalence has increased over the last 15 years, thus warranting revisiting the question of whether H9N2 exposures among people may influence heterosubtypic population immunity and overall influenza transmission dynamics.

In conclusion, our findings suggest that circulation of H9N2 in Vietnamese chickens is sufficiently widespread to be influencing the overall ecology and emergence potential of AIV, and we emphasize the need for further research to better understand possible interactions between co-circulating LPAI and HPAI subtypes. We fully support the call for continued vigilance in monitoring ongoing AIV evolution.

## Author disclosure statement

No competing financial interests exist.

## Figures and Tables

**Table 1:** H5 and H9 subtyping by RTPCR of H7F surveillance swabs, Oct.–Dec. 2014.

	AIV +	AIV + swabs screened (%)⁎	H5 + (%)	H9 + (%)	Dual H5 + H9 +	H5 + only	H5–H9 −
Bac Giang	230	97 (20.7)	2 (0.4)	62 (63.9%)	1	1	34
Bac Ninh	220	82 (17.5)	0	80 (97.6%)	0	0	2
Cao Bang	254	93 (19.9)	1 (0.2)	92 (98.9%)	1	0	1
Ha Giang	167	68 (14.5)	6 (1.3)	60 (88.2%)	3	3	5
Ha Noi	85	34 (7.3)	0	34 (100%)	0	0	0
Lao Cai	246	94 (20.1)	13 (2.8)	94 (100%)	13	0	0
Hưng Yên	5	0	0	0	0	0	0
Total	1207	468 (100)	22 (4.7)	422 (90.1)	18 (3.8)	4 (0.85)	42 (8.9)

⁎ Only swabs with AIV matrix gene RTPCR Ct < 26 were selected for screening.

## Appendix A Supplementary data

Supplementary Table S1 Molecular markers of virulence, airbourne transmission, drug resistance and mammalian adaption within Vietnamese H9N2 viruses: (a) hemagglutinin (segment 4); (b) NA gene (segment 6); (c) PB2 and PB1 (internal gene segments 1 and 2); (d) PA, PA-X, and NP (internal gene segments 3 and 5); (e) M1, M2, and NS1 (internal gene segments 7 and 8). All viruses were influenza A/chicken/Vietnam, and are indicated by their unique sequence identifiers. Reference viruses A/Lengshuitan/11197/2013 and A/Zhongshan/201501/2015 were the most closely related human isolates with whole genome sequences available (doi:10.1002/jmv.24231). ‘n/a’ indicates sequence data was not available for those regions. Supplementary Table S2 Assession numbers for Genbank submissions. Supplementary Table S3 Primer/probe sequences used for molecular screening. Image 1

Supplementary Appendix Modeling approaches to estimating prevalence from pooled samples (A) and interactions between subtypes (B). Supplementary Appendix
