# Supplementary material for: Prevalence and diversity of H9N2 avian influenza in chickens of Northern Vietnam, 2014
Source: Infect Genet Evol. 2016 Oct;44:530–40. doi: 10.1016/j.meegid.2016.06.038 (PMC5036934; doi:10.1016/j.meegid.2016.06.038)
Supplement: Supplementary file 1 — Supplementary Table S1 Molecular markers of virulence, airbourne transmission, drug resistance and mammalian adaption within Vietnamese H9N2 viruses: (a) hemagglutinin (segment 4); (b) NA gene (segment 6); (c) PB2 and PB1 (internal gene segments 1 and 2); (d) PA, PA-X, and NP (internal gene segments 3 and 5); (e) M1, M2, and NS1 (internal gene segments 7 and 8). All viruses were influenza A/chicken/Vietnam, and are indicated by their unique sequence identifiers. Reference viruses A/Lengshuitan/11197/2013 and A/Zhongshan/201501/2015 were the most closely related human isolates with whole genome sequences available (doi:10.1002/jmv.24231). ‘n/a’ indicates sequence data was not available for those regions. Supplementary Table S2 Assession numbers for Genbank submissions. Supplementary Table S3 Primer/probe sequences used for molecular screening. [file mmc1.docx]

Table S1. Molecular markers of virulence, airbourne transmission, drug resistance and mammalian adaption within Vietnamese H9N2 viruses: (a) hemagglutinin (segment 4); (b) NA gene (segment 6); (c) PB2 and PB1 (internal gene segments 1 and 2); (d) PA, PA-X, and NP (internal gene segments 3 and 5); (e) M1, M2, and NS1 (internal gene segments 7 and 8). All viruses were influenza A/chicken/Vietnam, and are indicated by their unique sequence identifiers. Reference viruses A/Lengshuitan/11197/2013 and A/Zhongshan/201501/2015 were the most closely related human isolates with whole genome sequences available (doi:10.1002/jmv.24231). ‘n/a’ indicates sequence data was not available for those regions.

Table S1. (b) NA gene (segment 6).

Table S1. (c) PB2 and PB1 (internal gene segments 1 and 2).

Table S1. (d) PA, PA-X, and NP (internal gene segments 3 and 5).

Table S1. (e) M1, M2, and NS1 (internal gene segments 7 and 8).

Table S2. Sequence identifiers and Genbank Accession #s. (still pending! We have just attempted submission to Genbank/NCBI again, June16. We hope to provide access# before the July13 deadline of submitting revisions.)

Table S3. Primer/probe sequences used in this study for molecular screening.
