# Supplementary material for: Prevalence and diversity of H9N2 avian influenza in chickens of Northern Vietnam, 2014
Source: Infect Genet Evol. 2016 Oct;44:530–40. doi: 10.1016/j.meegid.2016.06.038 (PMC5036934; doi:10.1016/j.meegid.2016.06.038)
Supplement: Supplementary Appendix — Modeling approaches to estimating prevalence from pooled samples (A) and interactions between subtypes (B). [file mmc2.pdf]

**Supplementary Appendix:** Description of modeling approaches to prevalence estimation and assessment of subtype interactions.

## A – Maximum-likelihood estimation of the prevalence from pooled samples

Consider *p* the true unknown prevalence in a given location over a given period of time and *n* the number of samples per pool. Then the probability that one pool of samples is positive reads

$$1-(1-p)^n$$

Consider now that *k* is the number of pools analyzed and $P_i$ is a binary variable equal to 1 if the pool is positive and 0 if it is negative. The likelihood function of the true prevalence *p* then reads

$$L(p)=\mathcal{B}\left(\sum_{i=1}^{k}P_i,k,1-(1-p)^n\right)$$

where *B* is the binomial density function. The true prevalence *p* was estimated by maximizing *L(p)*.

## B – Testing the interaction between two pathogens

We here assume that the prevalence of a pathogen is proportional to its force of infection. This assumption is fair as long as the pool of susceptible is not a limiting factor but becomes false when time and/or infectiousness tend toward infinity. Let's call *p* and *q* the probabilities of infection by pathogens A and B. Taking inspiration from the population genetics expression of the linkage disequilibrium between two loci, we model the interaction between the two pathogens by a parameter *D*. Thus, in one sample, the probabilities of (i) absence of infection, (ii) infection by A only, (iii) infection by B only and (iv) infection by A and B at the same time read:

$$\begin{aligned}p_0&=(1-p)(1-q)+D\\p_A&=p(1-q)-D\\p_B&=(1-q)p-D\\p_{AB}&=pq+D\end{aligned}$$

respectively. With *n*>1 samples per pool, the above probabilities become, at the level of poolled samples:

$$p_0 = (1-p)^n(1-q)^n + D$$

$$p_A = (1-p)^n \sum_{k=1}^{n-1} \binom{n}{k} q^k (1-q)^{n-k} - D$$

$$p_B = (1-q)^n \sum_{k=1}^{n-1} \binom{n}{k} p^k (1-p)^{n-k} - D$$

$$pAB = 1 - p_0 - p_A - p_B - p_{AB}$$

From these probabilities we can express the following likelihood function

$$L(p,q,D) = \mathcal{M}((N_0, N_A, N_B, N_{AB})^T, (p_0, p_A, p_B, p_{AB})^T)$$

where *M* is the multinomial density function and $N_0$, $N_A$, $N_B$, and $N_{AB}$ are respectively the observed numbers of pools with no infection at all, infection with A only, infection with B only, and infection with A and B at the same time. The significance of interaction between the two pathogens is then tested by a likelihood ratio test comparing a model $H_0$ imposing $D = 0$ and a model $H_1$ for which this constraint is relaxed.
